# Hand-Powered Elastomeric Pump for Microfluidic Point-of-Care Diagnostics

**DOI:** 10.3390/mi11010067

**Published:** 2020-01-07

**Authors:** Gangadhar Eluru, Jayesh Vasudeva Adhikari, Priyalaxita Chanda, Sai Siva Gorthi

**Affiliations:** Optics and Microfluidics Instrumentation Lab, Department of Instrumentation and Applied Physics, Indian Institute of Science, Bangalore 560012, India; eluru.gangadhar@gmail.com (G.E.); jayeshadhikari@gmail.com (J.V.A.); priyalaxitachanda@gmail.com (P.C.)

**Keywords:** micropump, point-of-care diagnostics, microfluidics, elastomeric pump, passive pumping

## Abstract

The pumping of fluids into microfluidic channels has become almost an unavoidable operation in all microfluidic applications. Such a need has seen an outburst of several techniques for pumping, out of which the majority of techniques involve complicated fabrication, as they require the introduction of electrodes, valves, piezoelectric materials, acoustic transducers, etc., into the microfluidic device. In addition to the complexity, this also escalates the cost incurred per device. Further, the use of stable external power supplies to produce such a pumping action adds to the bulkiness of the pumps, making them unsuitable for point-of-care diagnostic (POCD) applications. This paper reports a technique of pumping that is simple to realize and does not require external electric/magnetic power, but exploits the elastic properties of materials to achieve the pumping action. This mechanism of pumping ensured the cost per pump to less than 4 USD and can be used for at least 500 times. Several simulations, validation, and characterization experiments were performed on the developed pump to establish its functionality and suitability for use in POCD applications.

## 1. Introduction

Testing at point-of-care is a demanding, promising, and rapidly growing areas in healthcare sector, as it avoids sample transport to the laboratory and the need for manual preparation of samples, thereby providing the time advantage for taking urgent decisions on further diagnostic and therapeutic procedures. Microfluidic approaches with the unique advantages they offer, such as consumption of low sample and reagent volume, compactness, automation, and the ability to achieve integration with other microfluidic technologies leading to lab-on-chip are showing a promising future in the realization of such POCD devices. As microfluidics deals with handling fluids ranging from a few pico- to micro-liter volumes per second, it becomes almost incumbent on its part to have a fluid handling system that can deliver such small quantities of fluids. Efforts to realize such a fluid handling system have led to the development of several varieties of pumps that leverage various properties of the sample fluid and/or the actuating element such as electrical, mechanical, electro-mechanical, thermal, chemical, and pneumatic [1,2,3,4,5,6].

The pumps used in microfluidic applications can be broadly classified into two categories, viz., (1) micro pumps and (2) macro pumps. As per the convention used in microelectromechanical systems, pumps fall under the category of micropumps when the prominent feature size of the actuating element is of the order of 100 μm or less, otherwise considered as macropumps. Micropumps can be further sub-divided into mechanical displacement pumps and energy transfer pumps. Mechanical displacement pumps rely on the oscillatory mechanical motion of diaphragms or flaps for pumping the fluid, which in turn can be achieved through actuation mechanisms such as mechanical, piezoelectric, thermal, and pneumatic [7,8,9,10,11], whereas energy transfer pumps transfer the energy (electric, magnetic, and acoustic) directly to the sample fluid leading to fluid flow. Examples of this category include electrohydrodynamic, magnetohydrodynamic, electroosmotic, electrochemical, and ultrasonic pumps [12,13,14,15,16,17,18,19]. Both varieties of pumps have their own advantages and disadvantages. Mechanical pumps can offer larger back pressures and higher flow rates compared to energy transfer pumps; however, they are complex in fabrication and integration, produce pulsating flow, and require external power to operate. The energy transfer pumps offer no moving parts and can be integrated with other lab-on-chip devices with relative ease, while being able to generate fluid flow continuously and precisely. The major drawbacks associated with energy transfer pumps are their requirement of stable external power supplies that are bulky and expensive, inability to produce larger back pressures and flow rates, and complexity in fabrication. Apart from the above-mentioned drawbacks, the major disadvantage associated with either category of techniques is biological cell incompatibility, as they either reduce the cellular viability or cannot produce a pumping action on biological fluids. However, so far, none of these micropumps were able to address the true needs of POCD devices in terms of overall compactness, simplicity in fabrication and integration, smaller or no power consumption, low cost, and bio-cellular compatibility.

Those pumps that do not fall under the definition of micropumps can be categorized as macropumps. The most common macropumps that are in use in laboratory settings are syringe pumps. These pumps offer very stable flow rates, provide flexibility to vary the flow rates, and are compatible with biological cells. However, they are bulky and quite expensive, thereby making them not suitable for POCD devices. A compromise between cost, bulkiness, and complexity in fabrication and functionality seems to offer a promising solution for the development of a pump that can potentially make the realization of microfluidic POCD devices a reality. Even though the proposed technique of pumping in this paper is more general, it keeps in view the POCD applications such as whole blood counting and urinalysis that can be performed using imaging flow cytometry throughout the subsequent discussions.

Few micropumps that are self-powered and easy to fabricate have been proposed but their flow rates of operation are either too small to be practical for many of the cell counting diagnostics or difficult to control from application to application [20,21,22,23,24]. Our proposed technique differs from them as it can offer a wide variety of flow rates and offers ease in controlling the flow rates depending on the application. In addition, our technique can provide a constant flow rate of operation if an appropriate material is chosen, even though it is not demonstrated in this paper (refer to Section 3.7).

This work presents a pump that operates on the elastic properties of an elastomeric block in relaxation when it is released from compressive stress. This pump does not fall under the category of micropumps as its functional dimension is in millimeters instead of submillimeter. However, the overall design of the pump is compact and is easily portable. It is highly cost-effective (less than 4 USD per pump), biological cell compatible, and can be used directly with traditional ways of introducing a sample into microfluidic devices using regular syringes. Ease in replacing the elastomeric block provides the ability to change the flow rate, thereby making it highly versatile in use for POCD applications. This pump, once fabricated, can be reused at least 500 times without degradation in the quality of pumping, thereby ensuring its use for multiple tests prior to disposal. In addition, this pump from its fabrication perspective also serves as an excellent example for frugal innovation, as most of its parts are taken from the readily available components in the market. The proposed pump principle and design seem to offer an alternate solution for the pumping of microfluidic POCD devices. In the following sections, the principle and construction of pumps, their characterization and validation experiments, and their consequent implications are discussed.

## 2. Materials and Methods

### 2.1. Design and Operating Principle

**Figure 1:**
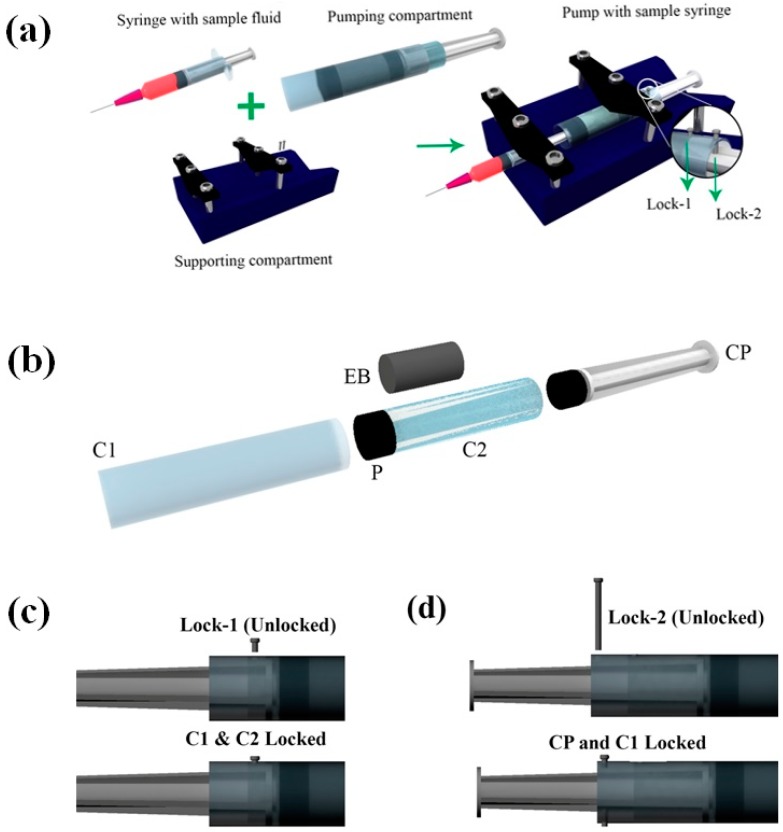
Schematics illustrating the various components of the pump. (**a**) Schematic of the pump and its various sub-compartments. Inset shows the locks used for locking and unlocking C1, C2, and CP. (**b**) Exploded view of the pumping compartment. (**c**,**d**) Schematics illustrating the locking and unlocking mechanism for locks 1 and 2, respectively. C1—large cylindrical tube, C2—small cylindrical tube, P—piston rubber, EB—elastic block, CP—compressor. Note: The direction of viewing for the figures (a,b) is in the opposite direction to that of (c,d).

Figure 1a shows the schematic of the pump developed. The pump design can be broadly divided into two parts, namely pumping compartment and supporting compartment. The supporting compartment serves the purpose of holding steadily the pumping compartment and the syringe with sample fluid as shown in Figure 1a. The pumping compartment consists of one large cylindrical tube that is open at both ends (C1), a small cylindrical tube (C2) that is closed at the front end with a piston rubber (P), an elastic block (EB), a compressor (CP), and a locking–unlocking mechanism. The respective arrangement is shown in Figure 1b,c. The elastic block EB gets into the smaller cylindrical tube C2. C2 lies inside the larger tube C1 and is free to move along the walls. C2 can be locked or unlocked to C1 using a small pin that goes through aligned holes punched to the two tubes (lock-1). At the rear side of the EB lies the compressor. This compressor CP can be locked to C1 using lock-2 that employs a similar mechanism as that of lock-1. On the front side of P lies the piston of the syringe that contains the sample fluid to be pumped.

EB has a diameter of *D_e_* and height *h_e_*, both being smaller than that of C2. CP has an outer diameter matching the inner diameter of the C2. The pump in its non-pumping condition will have C2 locked with respect to C1, while freely allowing CP to move. The CP, when pushed against the EB and locked with respect to C1, will lead to the development of compressive stresses within EB.

**Figure 2:**
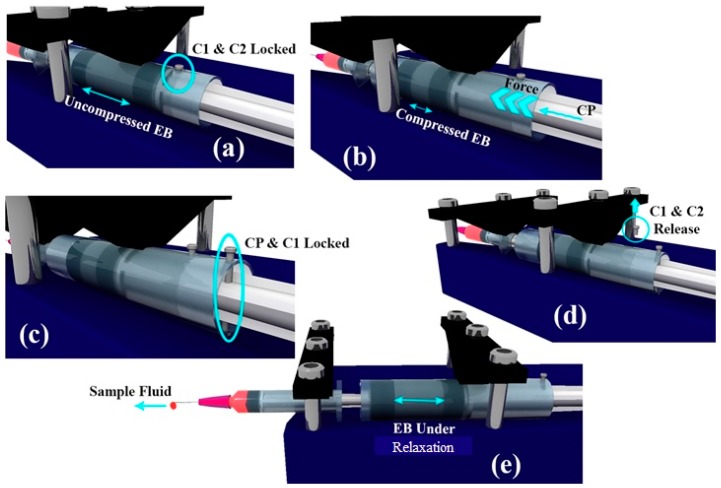
Schematic depicting the working of the pump. (**a**) C1 and C2 are locked using pin. (**b**) EB is compressed using CP. (**c**) CP and C1 are locked. (**d**) C1 and C2 are released thereby initiating the pumping action. (**e**) Image illustrating the pumping action while EB is relaxing.

If the locking between C2 and C1 is released, EB pushes C2 in the direction of the sample syringe piston to relieve the stored compressive stresses within the EB. This, in turn, pushes the sample syringe piston leading to the pumping of the sample fluid into the microfluidic device connected. The schematic depicting the working of the pump is shown in Figure 2.

### 2.2. Theory and Modeling

The force applied by the elastic block on the elastic container at any moment is given by the product of stress (*St*) the elastic block is experiencing and its area of cross-section (π × *D_e_*^2^/4). Presuming negligible frictional forces, the same force gets transferred to the sample fluid inside the syringe. Hence, the pressure acting on the sample fluid is given by:

(1) $$P=St\times \left(\pi \times D_{e}^{2}/4\right)/\left(\pi \times D_{s}^{2}/4\right)$$

where *D_e_* is the diameter of the elastic block, *D_s_* is the sample syringe inner diameter, and *St* is the stress that the elastic block is experiencing. This can be further simplified as:

(2) $$P=St\times \left(\frac{D_{e}^{2}}{D_{s}^{2}}\right)$$

If the syringe is attached to a microfluidic device, the overall hydrodynamic resistance the sample fluid feels for its flow is a combination of resistances offered by the syringe needle, tubing, and the microfluidic device. Typically, the microfluidic device hydrodynamic resistance (*R_h_*) will be much larger than the hydrodynamic resistances of the syringe needle and tubing, hence it can be taken as the effective resistance the fluid experiences for its flow. Whereas, the fluid at the inlet experiences a pressure that is due to the elastic block and the atmosphere. Presuming the device outlet stays at the same height as that of the inlet (which is typically the case), the outlet also feels the same quantity of atmospheric pressure. Hence, the net pressure drop acting on the sample fluid is only due to the elastic block and is given by Equation (2).

**Figure 3:**
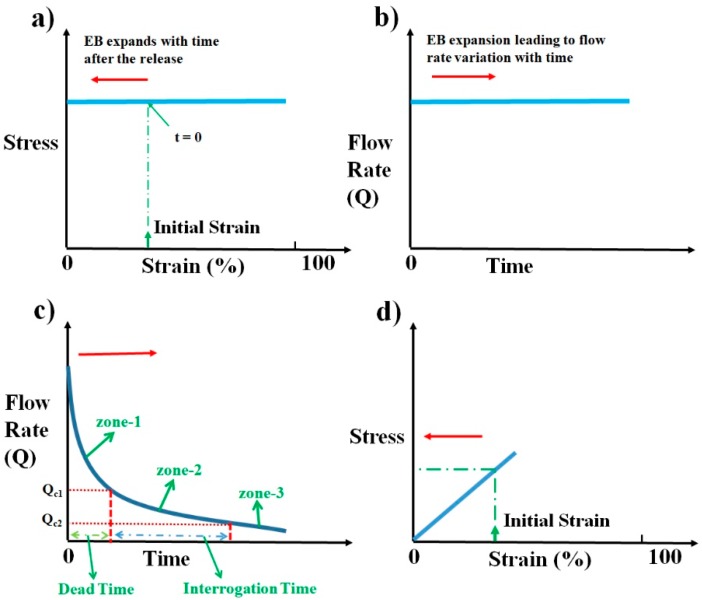
Schematics illustrating the flow rate variation with time and the respective stress–strain behavior desired from an elastomeric block. (**a**) Schematic of ideal stress–strain curve desired from an elastic material to produce a constant flow rate throughout the relaxation duration of the elastic block. (**b**) Schematic representing the expected flow rate variation with time. (**c**) Schematic illustrating the ideal flow rate characteristics desired for a pump that is suited to microfluidic point-of-care diagnostic (POCD) devices. (**d**) Schematic illustrating the proposed stress–strain curve to obtain the desired flow rate characteristics in (c). Arrow indicates the time evolution of strain during the operation of pump in (a,d), whereas it indicates the respective time evolution of flow rate with time in (b,c).

As the elastic block relaxes, depending upon the nature of elastic material, the stress the block exerts on the sample syringe changes. If the elastic block has stress–strain characteristics as shown in Figure 3a, irrespective of the initial strain induced, it will produce constant stress throughout the relaxation period. The pressure drops thereby produced across the device will be constant as given by Equation (2). Under constant pressure across the device, we can presume flow becomes steady quickly (≈sec) as compared to the time duration of operation (≈min) and the flow rate can be computed as per the steady-state Equation (3).

(3) $$Q=\frac{P}{R_{h}}=St\times \left(\frac{D_{e}^{2}}{D_{s}^{2}}\right)/R_{h}$$

As *D_e_*, *D_s_,* and *R_h_* are constants for a given device, the sample syringe, and pump, the flow rate *Q* at any moment is dependent upon the elastic stress *St* that the block exerts on the fluid, as shown in Figure 3b. This constant flow rate of pumping is the standard laboratory process followed in microfluidics using pumps such as syringe pumps. 

Even though this is what is being followed, it is not always a desired way of pumping from the perspective of gaining a time advantage while performing microfluidic POCD tests. A typical microfluidic POCD test involves the pumping of the sample fluid into the microfluidic device through the tubing. After the sample fluid reaches the microfluidic device, sample interrogation starts. This time lag between the start of the pumping and the sample fluid reaching the microfluidic device is the dead time involved in the diagnosis. This dead time is one of the critical times to be optimized to have an overall shorter duration of operation for any microfluidic POCD device. As an illustration, for imaging flow cytometric applications, the typical flow rate of operation will be around 100 µL·h^−1^ [25]. For a polyethylene tubing with an inner diameter of 0.38 mm and a length of 10 cm, the dead time of operation is around 7 min, which is very huge compared to the requirement of having the overall time of diagnosis to within 2–3 min. This clearly shows that providing a constant flow rate of operation as is conventionally followed in laboratory settings may not be an advantageous way of pumping from the perspective of overall diagnosis time. One of the ways to reduce this dead time is to have a very high flow rate of pumping at the beginning of pump operation, which continuously decreases to a lower desired range of flow rates as shown in Figure 3c. This high flow rate of operation ensures that the sample fluid crosses the tubing quickly and reaches the microfluidic device. Continuous reduction in flow rate from the initial value ensures that the sample fluid continues to decelerate and reaches the optimum flow rate desired for imaging when it reaches the device. This dead time zone is represented as zone-1 in Figure 3c. Dead time can be decreased to lower values if the initial flow rate of operation is very large and the rate of reduction is very steep. This is one of the desired features of an ideal pump for microfluidic POCD devices. Once the sample reaches the device, the flow rate of operation should be either constant or slowly vary within a certain flow rate range to avoid any reduction in throughput. The reduction in throughput can happen if the flow rate varies widely to such an extent that either some of the sample particles move at a higher speed (flow rate > *Q_c_*_1_), thereby causing motion blur, or some move very slowly (flow rate < *Q_c_*_2_), such that images of the same particle are captured multiple times. This can be avoided if the flow rate varies within an optimum range (between *Q_c_*_1_ and *Q_c_*_2_) during the interrogation. This time zone of interrogation is represented as zone-2 in the schematic shown in Figure 3c. The time zone represented as zone-3 in Figure 3c is the flow rate variation with time after the interrogation, which is not a critical time zone to scrutinize. The flow rate within that zone can decrease, which is in natural tune with how the flow rate is changing since the start of pumping. This will help to smoothly stop the process of pumping. These desired flow rate behaviors can be achieved from this pump using an elastomeric block that shows a linear stress–strain plot, as shown in Figure 3d.

In general, most of the materials show a stress–strain behavior that is linear at lower values of compressive strain, as shown in Figure 3d. Such materials, when compressed and released, show a stress that keeps decreasing as the material relaxes toward its natural length. If such a material is used as an elastomeric block for the pump, it results in decreasing stress and decreasing pressure difference across the microfluidic device as the block relaxes. This results in a variable flow rate of pumping as a function of time. The schematic illustrating the flow rate variation with time is shown in Figure 3c. The exact solution for this problem requires solving a coupled Navier–Stokes Equation whose input pressure difference depends on the output flow rate, which is computationally highly expensive. For slow variations of pressure difference across the device, the flow can be presumed to reach steady-state instantaneously and applying the steady-state Equation (3) to compute the flow rate at every value of pressure difference across the device. The above process of simulating the flow would be a reasonable approximation to gain good insights into the qualitative behavior of the flow. 

So far, the discussion neglected the presence of frictional forces, which, in practice, cannot be avoided altogether in the working of the pump. The major source of the friction comes from the movement of the piston rubber P that is in contact with C1 and from the movement of the sample syringe piston into the sample syringe. Presuming the friction coefficients are similar in both cases (due to the similar materials used), the net frictional forces acting on the sample syringe piston in the direction opposite to its movement is given by:

(4) $$F_{fr}=\;\gamma \times \pi \times D_{pi}\times D_{pl}+\gamma \times \;\pi \times D_{s}\times D_{sl}$$

wherein the first term is a contribution coming from piston rubber P and the latter is due to the sample syringe piston. γ is the friction coefficient between the piston rubber P and C1, and the sample syringe piston rubber and sample syringe. *D_pi_* and *D_pl_* are the inner diameter of C1 and the length of the piston rubber P, respectively. *D_s_* and *D_sl_* are the inner diameter of the sample syringe and the length of the sample syringe piston rubber, respectively. Hence, the net pressure the *P_net_* and the fluid experiences and the associated flow rate *Q_eff_* are given by:

(5) $$P_{net}=P-F_{fr}/\left(\pi \times D_{s}^{2}/4\right)$$

(6) $$Q_{eff}=P_{net}/R_{h}$$

where *P* is given by Equation (2). Simulations were performed in MATLAB (R2014a) to determine the flow rate of pumping as a function of time as per the above model for experimentally used parameters. As the frictional coefficient γ is unknown, its value was chosen such that the simulated flow rate matches with experimental observations at 1 min from the start of pumping. 

Elastomers, such as polydimethylsiloxane (PDMS), possess a compressive stress–strain curve that is linear for smaller values of strain, like the one shown in Figure 3d. By varying the amount of curing agent that is added to the fixed quantity of liquid PDMS to facilitate polymerization, the stiffness of the resulting solid PDMS can be controlled. We express this variable amount addition of curing agent as the ratio of PDMS to curing agent in this paper. The choice of PDMS as EB is due to the wide stiffness range it can offer and its ease of availability and fabrication. To validate the proposed pumping mechanism and characterize the fabricated pump, experiments were conducted with red blood cell (RBC) samples. The use of red blood sample solution serves a dual purpose; the first being a method to estimate the flow rate, while the other demonstrates the biological compatibility. The procedure for EB fabrication using PDMS, cost analysis, sample preparation, and experimental procedure and analysis are detailed in the following sections.

### 2.3. Materials and Fabrication

Pumping compartment was mostly made using readily available materials in lab, and the suitable support structure (or support compartment) was designed using computer-aided design and drafting software, SolidWorks. The support structure design was exported as STL (Standard Tessellation Language) file and was sliced using MakerBot slicing software prior to giving this as an input to the 3D printer. The 3D printer (Wanhao Duplicator desktop 3D printer) that works on the principle of fused deposition modeling based dual extrusion was used to print the parts, with Polylactic acid as build material. Extruder temperature of 210 °C and build base temperature of 60 °C was maintained throughout the print.

**Figure 4:**
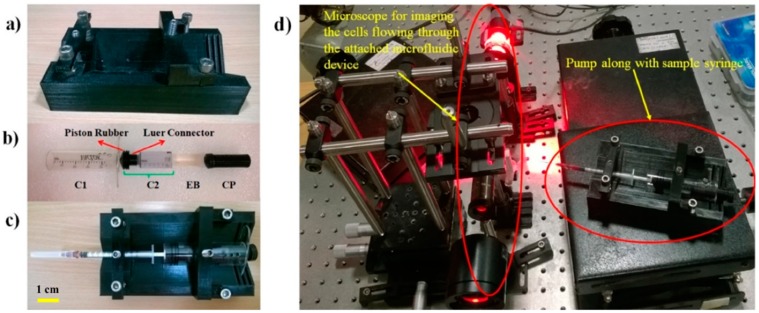
Prototype of the parts used in making the pump and characterization experimental set-up. (**a**) Support compartment. (**b**) Parts that make up pumping compartment. (**c**) Pump along with sample syringe. (**d**) Experimental set-up that was used for characterizing the performance of the pump.

The support structure consists of a base of dimension 8 cm × 15 cm with a V-shape groove (along the longer edge) and slots (along the shorter edge) designed to hold the cylinder (C1) and sample syringe firmly in place. The support structure also includes 2 V-shaped locks that were held together to the support base using standard M6 screws. This combination of V-shaped locks and base structure ensures easy and reproducible attachment of the pumping compartment to the support structure. The support structure built for the pump is shown in Figure 4a.

A 25 mL Vac-Lok syringe was cut (from the side of the needle) to produce a two-sided opening needed for the outer cylindrical tube C1. Two 3 mm holes were drilled at two different positions for locking C1 with C2 and CP. A 15 mL Vac-Lok syringe was cut from its rear end (away from the needle placing end) and was used as cylinder C2. A Luer connector was designed, 3D printed, and attached to the tip of C2. This Luer connector connects the tip of C2 to the piston rubber P that was taken from the 25 mL Vac-Lok piston to ensure the smooth movement of C2 inside C1. The compressor CP was designed and 3D printed. The CP diameter was matched to the inner diameter of C2 to facilitate smooth compression. The various parts involved in making up the pumping compartment are shown in Figure 4b. The whole of the pump along with the sample syringe is shown in Figure 4c.

Several single-use molds were prepared with an inner diameter of 13 mm and a height of 27 mm. These molds were used to prepare PDMS elastic blocks of varied stiffness. PDMS was mixed with curing agents in varying ratios (13:1, 15:1, 18:1, and 20:1), poured into the molds, and were cured overnight at 65 °C. The solidified PDMS blocks were carefully cut open from the molds without damaging them. The PDMS devices with three different hydrodynamic resistances used for testing were fabricated from the three pre-fabricated SU-8 Masters, using the standard process of soft lithography. The computed hydrodynamic resistances of the three Masters (say A, B, and C) are 2 × 10^13^ Pa·s/m^3^, 1.3 × 10^15^ Pa·s/m^3^, and 2.5 × 10^15^ Pa·s/m^3^, respectively. 

### 2.4. Cost Analysis 

The two Vaclock syringes that were used to prepare C1 and C2 cost about 2 USD. The material cost for printing the support compartment, Luer connector, and the CP is about 0.8 USD. The screws and pins used for locking C1, C2, and CP cost about 0.1 USD. The PDMS block material cost is about 1 USD. Hence, the total estimated material cost for the preparation of one pump comes around 3.9 USD, which is far less than any commercially available pumps for microfluidic applications. 

### 2.5. Sample Preparation 

Blood samples were collected from healthy subjects under the approval of Institute Human Ethical Committee (IHEC), Indian Institute of Science, Bangalore, India (The collection of blood sample was approved by the Institutional Human Research Ethics Committee of the Indian Institute of Science (28 October 2016) and was registered on the Institutional Human Ethics Committee Register (IHEC No: 19-28102016)). All research was performed in accordance with relevant guidelines/regulations and informed consent was obtained from all participants regarding the study. The RBC sample solutions were prepared from the fresh venous blood obtained from healthy subjects, which were already mixed with ethylenediaminetetraacetic acid (EDTA) anticoagulant during the collection process. RBCs were separated from the whole blood by centrifugation at 500× *g* for 5 min. Then, 10 μL of the resulting hematocrit was pipetted and diluted by adding 1 mL of phosphate buffer saline (PBS) (135 mM NaCl, 2.7 mM KCl, 10 mM Na_2_HPO_4_, 2 mM KH_2_PO_4_, and pH adjusted to 7.4) to prepare the RBC sample solution. This sample preparation process was used for all the experiments performed during the validation and characterization process. 

### 2.6. Stress–Strain Characterization 

Compressive stress–strain characterization of elastomeric blocks was performed using Instron-5967 tensile and compressive testing machine. The cylindrical PDMS blocks to be tested were placed appropriately inside the instrument and the compressive testing was done at a strain rate of 0.027 mm/s. The obtained load–displacement curves were used to plot the stress–strain curves.

### 2.7. Experimental Procedure 

The experimental procedure involves loading the sample syringe connected to a microfluidic device onto the pump that contains the elastic block, and subsequently compressing and releasing the block as described earlier, thereby leading to pumping action. The RBC sample solution that was pumped into the microfluidic device was imaged using a custom-built bright-field transmission microscope to determine the flow rate of pumping as a function of time. The experimental set-up consisted of a Thorlabs LED (630 nm), Olympus micro objective (40×, 0.75 NA), auxiliary optics to achieve Köhler illumination, a holder to hold the microfluidic device, and a high-speed imaging camera (Mikrotron Eosense, MC-1362), as shown in Figure 4d. 

### 2.8. Flow Rate Estimation Methodology 

The procedure that was followed for flow rate determination involves finding the velocity of RBC in the flow as a function of its position across the channel cross-section inside the microfluidic device and subsequently computing flow rate based on an analytical expression that relates flow rate with velocity at a particular position. Velocity determination requires the acquisition of an RBC image at least twice as it flows. To ensure this, the frame rate of the camera was chosen high enough (1030 frames per second) during the video capture.

As the RBC flows along the length of the channel (z-direction), let us say the first captured image of the RBC has coordinates (x, y, z). As the channel does not have any tilt in the xz-plane, the next image of the RBC will have the same x-coordinate *x*, but a different z-coordinate *z* + δ*z*. The difference in z-coordinates, divided by magnification *M*, and the time difference (δ*t*) between the two acquired images yields the velocity of the cell at time *t*, given by the expression:

(7) $$u{\left(x,y,z,t\right)}_{experimental}=\delta z/\left(M\times \delta t\right)$$

The time difference between the acquired images is equal to the inverse of the frame rate as two consecutive frames of a video were used for analysis. The steady-state analytical expression for fluid velocity at (x, y, z) inside a microfluidic channel of width *w* (along x-direction), height *h* (along y-direction), and length *L* is given by:

(8) $$u\left(x,y,z,t\right)=P_{eff}/\left(2\times \mu \times L\right)\{\left[{\left(h/2\right)}^{2}-y^{2}\right]-\overset{\infty }{\underset{0}{\sum }}a_{n}\times cos(2\times \lambda_{n}\times y/h)\times cosh(2\times \lambda_{n}\times x/h)\}$$

where $\lambda_{n}=\left(2n+1\right)\times \pi /2$, $a_{n}=h^{2}\times {\left(-1\right)}^{n}/[\lambda_{n}^{3}\times cosh(\lambda_{n}\times w/h)]$, and *z* is the direction of flow. Choosing the plane of observation to be close to the center of the channel, i.e., *y* = 0 and for *h* < *w*, we can approximate the above expression to first order, and can be written as:

(9) $$u\left(x,y=0,z,t\right)=P_{eff}/\left(2\times \mu \times L\right)\times [{\left(h/2\right)}^{2}-8\times h^{2}/(\pi^{3}\times cosh\left(\pi \times w/\left(2\times h\right)\right))]$$

Substituting the experimentally determined value of velocity from Equation (7) along with channel dimensions and the x-location of the cell, the analytical Equation (9), for velocity, yields the unknown *P_eff_*/(*μL)*. This computed value of the unknown is substituted in the following analytical Equation (10) to estimate the flow rate. This analytical expression for flow rate gives accurate results within 10% error for *h*/*w* ≤ 0.7 [26].

(10) $$Q=\left[P_{eff}/\left(\mu \times L\right)\right]\times \left(w\times h^{3}/12\right)\times \left[1-\left(192/\pi^{5}\right)\times \left(h/w\right)\right]$$

## 3. Results and Discussion

### 3.1. Variation of Flow Rate with Time 

**Figure 5:**
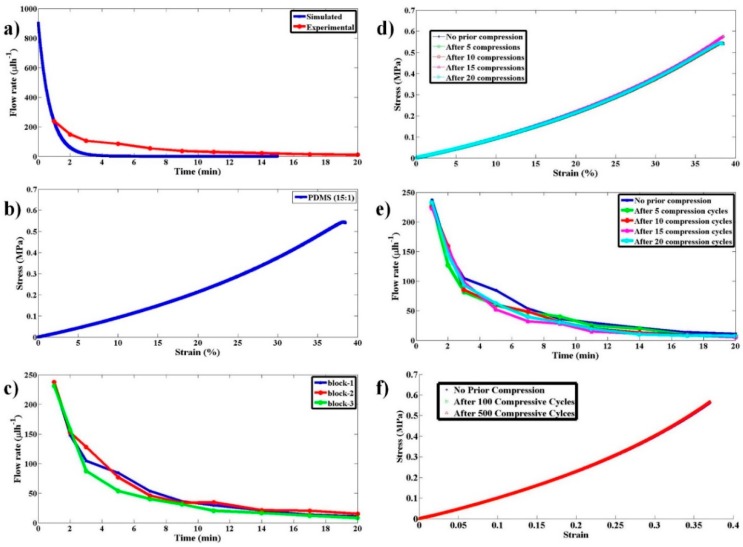
Studies on pump flow rate variation with time and its reproducibility and reusability as a multi-use pump. (**a**) Experimental and simulated flow rate as a function of time for the proposed pump when a PDMS block with 15:1 ratio was used. (**b**) The stress–strain curve for the PDMS block with 15:1 ratio. (**c**) Experimental flow rate as a function of time for the proposed pump when three PDMS blocks made under identical conditions, with 15:1 ratio, were used to test the reproducibility of the pump. (**d**) The stress–strain curve for the PDMS block with 15:1 ratio and have undergone 0, 5, 10, 15, and 20 compression-release cycles prior to using them for pumping. (**e**) Experimental data for flow rate as a function of time using the PDMS blocks that have undergone compression-release cycles as in (d). (**f**) The stress–strain curve for the PDMS block with 15:1 ratio and have undergone 0, 100, and 500 compression-release cycles prior to using them for pumping.

The elastomeric PDMS block (15:1) was compressed and released appropriately to provide a pumping action and the flow rate estimation was made by imaging human red blood cells in the flow. Instantaneous flow rates were computed as a function of time in a time window of 1 to 20 min, as shown in Figure 5a. The stress–strain curve for the PDMS (15:1) is shown in Figure 5b. Based on the theoretical model proposed and the experimental stress–strain curve, the flow rate variation with time was simulated and matched to the experimentally computed flow rate variation with time as, shown in Figure 5a. Qualitatively experimental flow rate variations match very well to that of the simulated variation. The discrepancy in quantitative results can be majorly attributed to the inaccuracy in approximating the time-evolving Navier–Stokes Equation to quasi-static steady-state approximation.

### 3.2. Repeatability and Reusability Tests 

To check the repeatability of the flow rate variation with time for an elastomeric block with a given stiffness, few PDMS blocks with PDMS to curing agent ratio of (15:1) were made and independent experiments were performed. The respective variation is shown in Figure 5c. It can be clearly observed that there is not much of a variation in flow rate from block to block so long as they are made with the same PDMS to curing agent ratio (same stiffness).

To validate the reusability of the pump for POCD applications, flow rate variation experiments with time were performed with blocks (15:1) that have undergone multiple compression and relaxation cycles. The variation of stress with strain and the flow rate with time are shown in Figure 5d,e, respectively.

To observe the behavior of the stress–strain curves under a large number of compressive-release cycles (0, 100, and 500), experiments were performed, and the results are shown in Figure 5f. The substantial overlap of the curves clearly shows that this pump produces the same flow rate variation even after several cycles (>500) of operation. This (reusability) is an important aspect to be characterized for evaluating the use of the pump for POCD applications as a single pump needs to be used for conducting a large number of tests. These properties clearly illustrate the robustness of the pump and its practical utility.

### 3.3. Feasibility of the Use of Pump for Cell Imaging in POCD Applications

**Figure 6:**
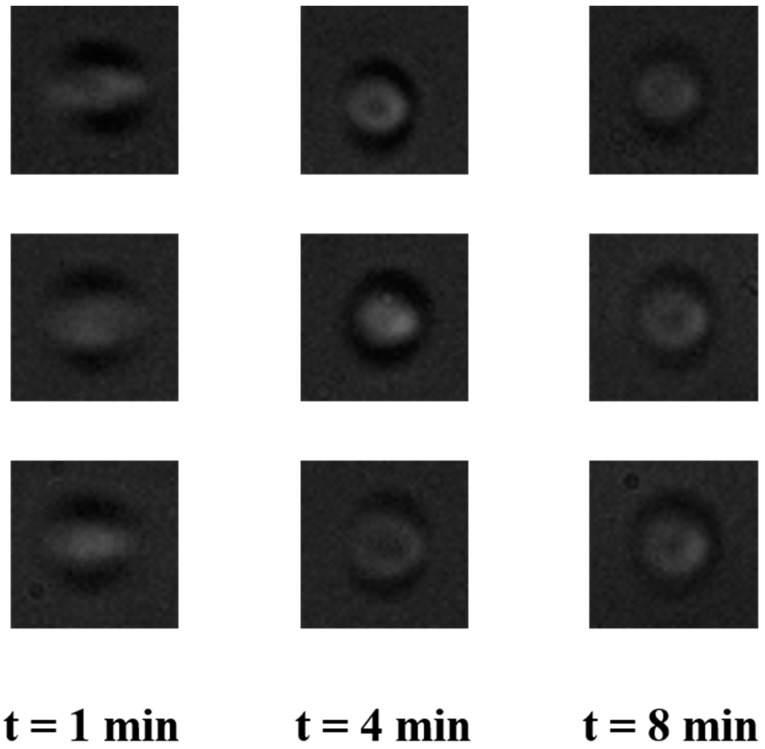
Representative images of red blood cells (RBCs) in the flow in a microfluidic device at different times after the start of pumping. The images are out of focus due to the larger depth of the channel that was used for imaging.

To validate the applicability of the use of the pump for imaging cells in the flow, RBCs were pumped through a microfluidic device and were imaged. Figure 6 shows the representative images of RBCs at different time intervals. As it is clear from Figure 6, the blur-free images of RBCs can be obtained around 4 min after the start of pumping, which agrees well with our hypothesis. This is a clear indication of the feasibility of the use of the proposed pump for POCD applications. 

### 3.4. Flow Rate Variation with Stiffness of the Elastomeric Material

**Figure 7:**
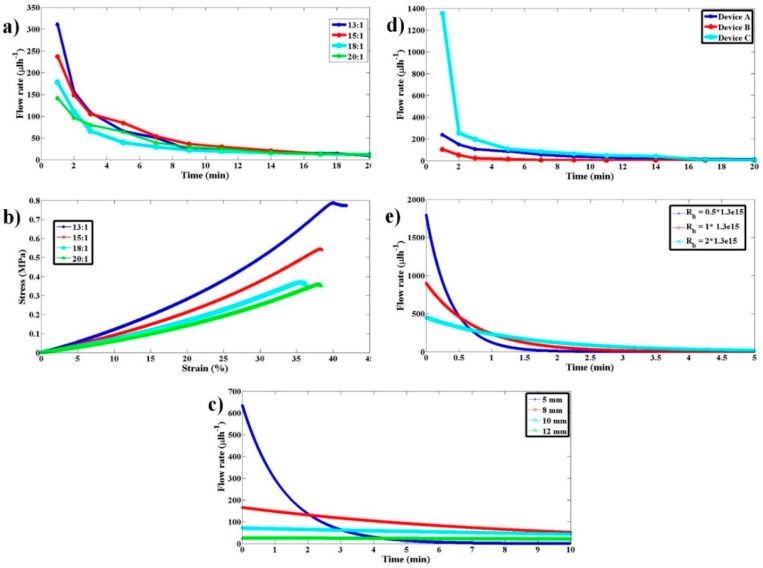
Flow rate variation characterization with respect to variations in PDMS stiffness, sample syringe inner diameter, and hydrodynamic resistance of the microfluidic device connected. (**a**) Experimental flow rate as a function of time for the proposed pump when PDMS blocks with 13:1, 15:1, 18:1, and 20:1 ratios were used. (**b**) The stress–strain curves for the respective PDMS blocks in (a). (**c**) Simulation results illustrating the dependence of flow rate on sample syringe inner diameter for 5, 8, 10, and 12 mm. (**d**) Experimental flow rate as a function of time for the proposed pump when a PDMS block with 15:1 ratio and different hydrodynamic resistance (Devices A, B, and C) were used. (**e**) Simulation results illustrating the flow rate dependence on microfluidic device hydrodynamic resistance.

The process of finding the flow rate variation with time was repeated for blocks with different Young’s Modulus (produced by varying the PDMS to curing agent ratio) and the respective variations are shown in Figure 7a. The stress–strain behavior of all these blocks is shown in Figure 7b. Figure 7a clearly shows us that the stiffer the block, the larger the flow rate it produces. As all POCD devices do not operate at the same flow rate, this pump offers flexibility in fetching the desired flow rate by choosing an appropriate stiffness of the block. Even though the flow rate differences due to different stiffnesses of the blocks were large at the beginning of pumping, as time passed, they were reduced. This is expected because as the blocks regain their original length with time, the stresses the blocks exert decreases leading to a decrease in the difference in stress among different blocks. This, in turn, leads to the decreased flow rates and the decreased difference in flow rates across the blocks (having different stiffness) with time. This will not be a problem so long as the POCD device is going to operate only for few minutes from the start of the pumping, as within that duration of operation blocks with different stiffness produce substantially different flow rates.

### 3.5. Variation of Flow Rate with Sample Syringe Inner Diameter 

Simulations were performed to understand the qualitative behavior of flow rate with time as the inner diameter of the sample syringe, *D_si_,* is varied. As the sample size increases, the stress that gets transferred onto the sample fluid decreases as 1/*D_si_*^2^ for a given amount force from the pump. This causes the flow rate to decrease by the same factor. For a given flow rate, the displacement of the sample syringe piston goes as 1/*D_si_*^2^. Hence, for a given force from the pump, the displacement of the sample syringe piston or to say the relaxation of the elastomeric block goes as 1/*D_si_*^4^. The faster the relaxation of the pump, the quicker the drop in flow rate with time. As the inner diameter of the sample syringe increases, the rate of relaxation of the pump decreases as 1/*D_si_*^4^, thereby leading to a more stable flow rate with time compared to using the smaller sample syringe. The simulated behavior of the same is shown in Figure 7c for *D_si_* of 5, 8, 10, and 12 mm. 

### 3.6. Variation of Flow Rate with Device Hydrodynamic Resistance

It is likely that different POCD devices offer different hydrodynamic resistances. To understand the dependence of flow rate on device hydrodynamic resistance three microfluidic devices (A, B, and C with different channel cross-sectional features) with computed hydrodynamic resistances 2 × 10^13^ Pa·s/m^3^, 1.3 × 10^15^ Pa·s/m^3^, and 2.5 × 10^15^ Pa·s/m^3^ were used, respectively. The pump comprised of EB with PDMS to curing agent ratio of 15:1. Experimental variations of flow rate with time and device hydrodynamic resistance are shown in Figure 7d. To understand the qualitative behavior, these flow rate variations with hydrodynamic resistance have been simulated, as shown in Figure 7e. The qualitative behavior of the experimental results is like that of the simulation results. The quantitative data mismatch for similar hydrodynamic resistance values across simulation and experiments is attributed to approximating the time-evolving Navier–Stokes Equation to quasi-static steady-state approximation. The qualitative behavior shows that the flow rate decreases as the hydrodynamic resistance increases. Having quantitative knowledge of flow rate variation with hydrodynamic resistance and EB stiffness will provide guidelines for choosing a suitable EB for a given device and desired flow rate. 

### 3.7. Discussion on the Production of Constant Flow Rates

Even though the paper emphasized the importance of variable flow rates of operation so far, there might be some POCD applications that require a constant flow rate during the investigation of the sample. This requirement of constant flow rates may arise if the interrogation duration is much larger compared to the transit time in the tubing, which may happen in devices used for rare cell detection such as circulating tumor cells in cancer. This could be achieved by suitably choosing an elastomeric block that shows a stress–strain curve a portion of which is (during the operation of the pump) similar to Figure 3a. An example of such an elastic material is high-density open-structured polyurethane, which shows a linear stress–strain behavior for smaller strains and produces almost constant stress for moderate strain [27]. We could not demonstrate this constant flow rate of pumping using polyurethane foams (that could be procured by us) due to the smaller stress produced by the foams compared to the opposing frictional forces inside the pump. However, this does not eliminate the possibility of such a constant flow rate production using this pump principle.

## 4. Conclusions

In conclusion, this paper reports a simple, compact, reusable, cost-effective, and electric (magnetic) power-free pump that operates on the underlying principle that any elastic material under compression, if released at one end, will lead to the pushing of that boundary. This push is leveraged for the pumping of the sample fluid into the microfluidic device. The pump’s working principle was demonstrated, and its performance was characterized using the PDMS elastomeric blocks. Most of the materials needed for the construction of the pump have been taken from the readily available materials in the market and/or easily fabricable material in the lab making it an excellent example of frugal science. This has enabled the cost per pump to be less than 4 USD. The pumping mechanism was found to be bio-compatible as the pump does not disrupt the cell membranes of the red blood cells used as the sample solution. PDMS blocks with different stiffnesses were used to demonstrate the potential to change flow rates (150 μL·h^−1^–300 μL·h^−1^). Unchanging stress–strain behavior even after several compression and relaxation cycles clearly indicates its robustness and reusability for several hundred (>500) tests. The pump and its mechanism of pumping were modeled, and simulations were performed to understand the qualitative behavior of pumping. Simulations have further given insights into the behavior of flow with changing sample syringe diameter. As a summary, the pump offers the ability to produce desired flow rate variation with time for a given test/experiment, flexibility to change the flow rates at the beginning of its operation, suppleness in choosing the flow rate based on POCD device, the ability to reuse, and ease of fabrication at an ultra-low cost, thereby providing great promise toward the realization of point-of-care diagnostic testing devices using microfluidics.
